# Identification and Characterization of the *OCT4* Upstream Regulatory Region in *Sus scrofa*

**DOI:** 10.1155/2019/2130973

**Published:** 2019-03-12

**Authors:** Seung-Hun Kim, Kwang-Hwan Choi, Dong-Kyung Lee, Mingyun Lee, Jae Yeon Hwang, Chang-Kyu Lee

**Affiliations:** ^1^Department of Agricultural Biotechnology, Animal Biotechnology Major, Research Institute of Agriculture and Life Science, Seoul National University, Seoul 08826, Republic of Korea; ^2^Designed Animal & Transplantation Research Institute, Institute of Green Bio Science and Technology, Seoul National University, Gangwon-do 25354, Republic of Korea

## Abstract

*OCT4* plays pivotal roles in maintaining pluripotency during early mammalian embryonic development and in embryonic stem cells. It is essential to establish a reporter system based on the *OCT4* promoter region to study pluripotency. However, there is still a lack of information about the porcine *OCT4* upstream reporter system. To improve our understanding of the porcine *OCT4* regulatory region, we identified conserved regions in the porcine *OCT4* promoter upstream region by sequence-based comparative analysis using various mammalian genome sequences. The similarity of nucleotide sequences in the 5′ upstream region was low among mammalian species. However, the *OCT4* promoter and four regulatory regions, including distal and proximal enhancer elements, had high similarity. Next, a functional analysis of the porcine *OCT4* promoter region was conducted. Luciferase reporter assay results indicated that the porcine *OCT4* distal enhancer and proximal enhancer were highly activated in mouse embryonic stem cells and embryonic carcinoma cells, respectively. A comparison analysis of naïve and primed state marker gene expression in a dual-reporter assay showed that the expression levels of naïve and primed markers differed in fluorescence signal between high-expressing cells and low-expressing cells. Similar to *OCT4* upstream-based reporter systems derived from other species, the porcine *OCT4* upstream region-based reporter constructs showed exclusive expression patterns depending on the state of pluripotency. This work provides basic information about the porcine *OCT4* upstream region and various porcine *OCT4* fluorescence reporter constructs, which can be applied to study species-specific pluripotency in early embryo development and the establishment of embryonic stem cells in pigs.

## 1. Introduction

Proliferation and differentiation are characteristics of stem cells, which are valuable for their use in human regenerative medicine. Economical animals, especially pigs, are useful for the production of disease models and transgenic animals. There are two states of stem cells: naïve and primed [1, 2]. Mouse epiblast stem cells are in the primed state and do not produce chimera after blastocyst injection analysis. However, mouse embryonic stem cells can form chimera after blastocyst injection analysis; therefore, they are naïve [3, 4]. This is a higher pluripotent state, but it has never been reported in animals other than mouse. Although research on pluripotent stem cells is underway in pigs [5–9], no naïve pig embryonic stem cells have been reported.

Because embryonic stem cell populations are not homogenous, reporter systems are used to classify the status of stem cells and isolate cells in certain conditions. Although it is one of the most necessary parts of studying stem cells and pluripotency, the lack of such research remains a problem. *OCT4*, a pluripotent marker, is one of many pluripotency-related genes that have been studied as a reporter gene since it is only expressed in pluripotent cells [10]. The transcription factor *OCT4* is an important marker of undifferentiated status in early mammalian embryonic development and embryonic stem cells. It has a critical role as a central regulator in maintaining pluripotency and self-renewal. There are four conserved regions (CR1, CR2, CR3, and CR4) in the 5′ upstream regulatory region of various species [11]. Moreover, *OCT4* contains a core promoter and two conserved enhancers, the distal enhancer (DE) and proximal enhancer (PE) [12–14]. The two elements regulated by retinoic acid exist in the PE region. As such, the loss of occupancy in these elements is called PE1A and PE1B, respectively. An element called DE2A has a sequence similar to that of PE1A and exists in the DE region [15–17]. A study of a mouse *Oct4* upstream region model revealed that the two enhancer regions were activated differently. The DE region can regulate *Oct4* expression in mouse embryonic stem cells, germ cells, and inner cell mass cells, whereas the PE region can regulate *Oct4* expression in mouse epiblast stem cells and epiblasts [12, 14, 18].

The *Oct4*-based reporter system has already been developed in other species and is used to distinguish, separate, and identify pluripotent stem cells [19–22]. However, there has been a lack of information on the porcine *OCT4* upstream reporter system until now. Porcine *OCT4* upstream region-based GFP reporter systems were reported in 2011 [23, 24], and a dual-reporter system using GFP and RFP was reported in 2016 [25]. However, a luciferase assay, which is used for promoter analysis, was not conducted [17, 19, 20]. Thus, these studies were limited by the fact that the classification of cells using the reporter system was only based on cell morphology analysis.

In this study, we first conducted an analysis of the porcine *OCT4* upstream region. A luciferase assay was performed for functional analysis of the promoter region. Next, we used a porcine *OCT4* upstream-based dual-reporter system to determine the mRNA expression of cells that were separated by fluorescence-activated cell sorting (FACS). Through the above experiments, we confirmed that the cells could be separated by our reporter system.

## 2. Materials and Methods

### 2.1. Animal Welfare

The authors assert that all procedures that contributed to this work complied with the ethical standards of the relevant national and institutional guides on the care and use of laboratory animals. The care and experimental use of pigs were approved by the Institute of Laboratory Animal Resources, Seoul National University (SNU-140328-2).

### 2.2. Sequence Alignment of *Oct4* URS in Animals and Sequence-Based Analysis

A 3.5 kb porcine *OCT4* nucleotide sequence was aligned with the *Oct4* sequences of human, goat, rabbit, mouse, bovine, and vole from the University of California Santa Cruz (https://genome.ucsc.edu/). Computational analysis of DNA sequences was conducted using the DNA sequence-based analysis programs ALIGN Query, Clustal, and BlastN. Putative transcription factor binding sites (TFBS) from mouse and pig DNA sequences were identified and compared.

### 2.3. Construction of Porcine *Oct4* Upstream Region-Derived Reporter

**Figure 1:**
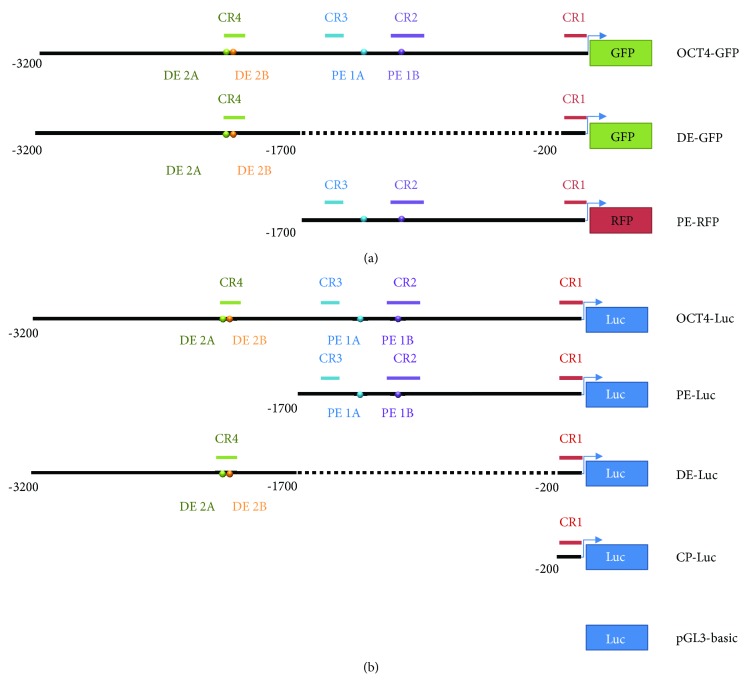
Schematic illustration of the constructed vector using the porcine *OCT4* promoter. (a) EGFP reporter vectors carrying different elements of the pig *Oct4* URS were constructed from a pEGFP-N1 vector. One RFP reporter vector carrying a 1.7 kb pig *Oct4* URS was constructed from a pDsRed2 vector. (b) Four constructs containing the different elements of the *OCT4* promoter in the pGL3-basic vector and the pGL3-basic vector alone as a control were transfected into cells as described in Materials and Methods. DE: distal enhancer; PE: proximal enhancer. The deleted regions are shown as dotted lines.

To generate the longest 3.2 kb *OCT4* upstream region-based GFP reporter system (OCT4-GFP), 3.2 kb of the *Oct4* regulatory region was inserted into a peGFP vector [23]. A 188,961 bp BAC clone (CH242-102G9) was used as sample DNA. It was used to construct a p*OCT4*-∆PE-eGFP vector (DE-GFP) containing CR1 and CR4 and a p*OCT4*-∆DE-DsRed2 vector (PE-RFP) containing CR1, CR2, and CR3 (Figure 1(a)). Based on this, four vectors were constructed to conduct luciferase analyses. The longest p*OCT4*-pGL3 basic vector (OCT4-Luc) contained all conserved regions, the p*OCT4*-∆PE-pGL3 basic vector (DE-Luc) contained CR1 and CR4, and the p*OCT4*-∆DE-pGL3 vector (PE-Luc) contained CR1, CR2, and CR3. Lastly, the p*OCT4*-CP-pGL3 basic vector (CP-Luc) contained only CR1 (Figure 1(b)).

### 2.4. Embryonic Stem Cell, Embryonic Carcinoma Cell, and Embryonic Fibroblast Culture

Embryonic stem cell culture media consisted of Dulbecco's minimum Eagle's medium (DMEM) (WELGENE) supplemented with 15% fetal bovine serum (FBS), 2 mM GlutaMAX, 0.1 mM *β*-mercaptoethanol, 1× minimum essential medium (MEM) nonessential amino acids (Gibco), 1× antibiotic/antimycotic, and 1000 units/mL of leukemia inhibitory factor (LIF; Millipore, MA, USA). Embryonic carcinoma culture and embryonic fibroblast media consisted of DMEM (WELGENE) supplemented with 10% FBS, 2 mM GlutaMAX, 0.1 mM *β*-mercaptoethanol, 1× MEM nonessential amino acids (Gibco), and 1× antibiotic/antimycotic. The media were changed every day, and all cells were cultured under humidified conditions with 5% CO_2_ at 37°C. When colonies of mouse embryonic stem cells and mouse embryonic carcinoma cells were ready for passaging, the cells were subcultured using trypsin.

### 2.5. Luciferase Activity Assay

**Figure 2:**
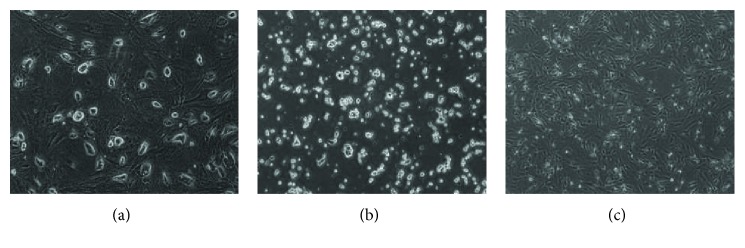
Mouse embryonic stem cells, embryonic carcinoma cells, and embryonic fibroblasts used in the experiments: (a) ES-E14TG2a mouse embryonic stem cells; (b) P19 mouse embryonic carcinoma cells; (c) mouse embryonic fibroblasts.

ES-E14TG2a mouse embryonic stem cells, P19 mouse embryonic carcinoma cells, and porcine embryo fibroblast cells were plated into six-well plates (Nunc) (Figure 2). Each reporter construct was transfected into cells using the Lipofectamine 3000 reagent (Invitrogen) according to the manufacturer's protocol. Cell lysate was prepared 48 h after transfection. Luciferase activity was detected using a Luciferase Assay System (Promega). Transfection of the pGL3-basic vector (Promega), which does not contain inserted DNA, was used as a control to determine the background level of luciferase activity.

### 2.6. Fluorescence-Activated Cell Sorting

ES-E14TG2a mouse embryonic stem cells were plated into six-well plates (Nunc). Each reporter construct was transfected into cells using the Lipofectamine 3000 reagent (Invitrogen) according to the manufacturer's protocol. After 48 h, embryonic stem cells were suspended in fluid by trypsin treatment. The cells were separated according to the degree of fluorescence signal using a FACSAria II (BD Biosciences).

### 2.7. Analysis of mRNA Expression by Quantitative Real-Time PCR

To verify the gene expression levels in pluripotent cells, we performed quantitative real-time PCR (qPCR). Total RNA from individual samples was extracted using a Dynabeads mRNA DIRECT kit (Life Technologies) according to the manufacturer's instructions. cDNA synthesis was performed using a High-Capacity RNA-to-cDNA (cDNA reverse transcription) kit (Applied Biosystems, Foster City, CA, USA) according to the manufacturer's instructions. Briefly, at a final volume of 20 *μ*L, cDNA synthesis was performed at 37.5°C for 60 min, and samples were subsequently incubated at 95°C for 5 min to activate the reverse transcription reaction. Synthesized cDNA samples were stored at −80°C until subsequent use. qPCR was performed using an ABI 7300 Real-Time PCR System (Applied Biosystems). A DyNAmo HS SYBR Green qPCR Kit (Thermo Scientific, Rockford, IL, USA) was used for real-time quantification of the PCR products. For amplification, 0.1 *μ*M of each primer listed in Supporting Information Table 1 and 0.5 *μ*L of cDNA were added to a 10 *μ*L reaction mixture. The reactions were performed under the following conditions: 1 cycle at 95°C for 10 min, 40 cycles at 95°C for 15 s, and 60 s at the annealing temperature. The dissociation curve was analyzed to confirm the specificity of the PCR product. *ACTB* was used as a control gene to determine the relative quantity. All genes were measured in triplicate, and the relative expression ratios were analyzed using the 2^-ΔΔCt^ (threshold cycle) method [26].

### 2.8. Statistical Analysis

All data were analyzed using the GraphPad Prism statistical program (GraphPad Software, San Diego, CA, USA). qPCR data were analyzed using analysis of variance and Fisher's least significant difference. All data are reported as the means ± standard error of the mean. *P* values less than 0.05 were considered statistically significant.

## 3. Results

### 3.1. Sequence Analysis of the *Oct4* 5′ Upstream Region

**Figure 3:**
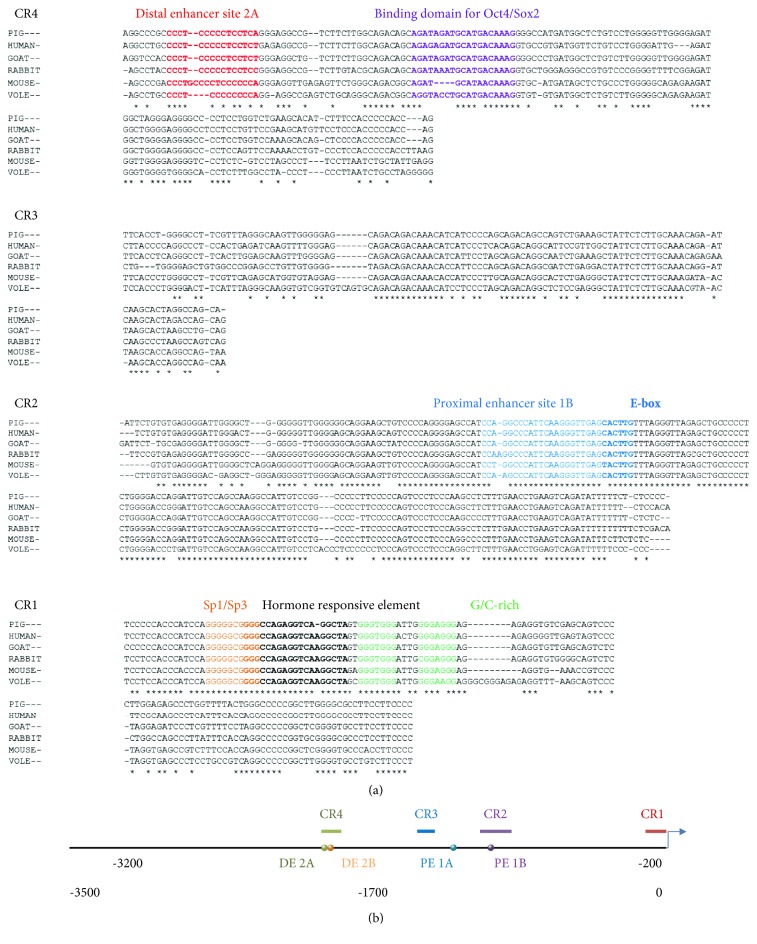
Alignment of porcine *OCT4* transcriptional regulatory regions with those of five other mammalian orthologs and schematic representation of the porcine *OCT4* transcriptional regulatory region. (a) Conserved region 4 (CR4) contains distal enhancer site 2A (DE2A) and the binding domain for *Oct4* and *Sox2* (*Oct4*/*Sox2*). Conserved region 3 (CR3). Conserved region 2 (CR2) contains proximal enhancer site 1B (PE1B). Conserved region 1 (CR1) contains the Sp1/Sp3 site, hormone responsive element (HRE), and three G/C-rich sites. The asterisks show the nucleotides that are identical among the six species. (b) The black line represents the 3.5 kb upstream region (located at positions -3500/-1) of *OCT4* in pig. The four upper lines of CR1, CR2, CR3, and CR4 represent the four conserved regions, respectively. The circles show the locations of distal enhancer site elements 2A (DE2A) and 2B (DE2B) and the proximal enhancer site elements 1A (PE1A) and 1B (PE1B). The arrow indicates the translational initiation site of the *OCT4* gene.

**Figure 4:**
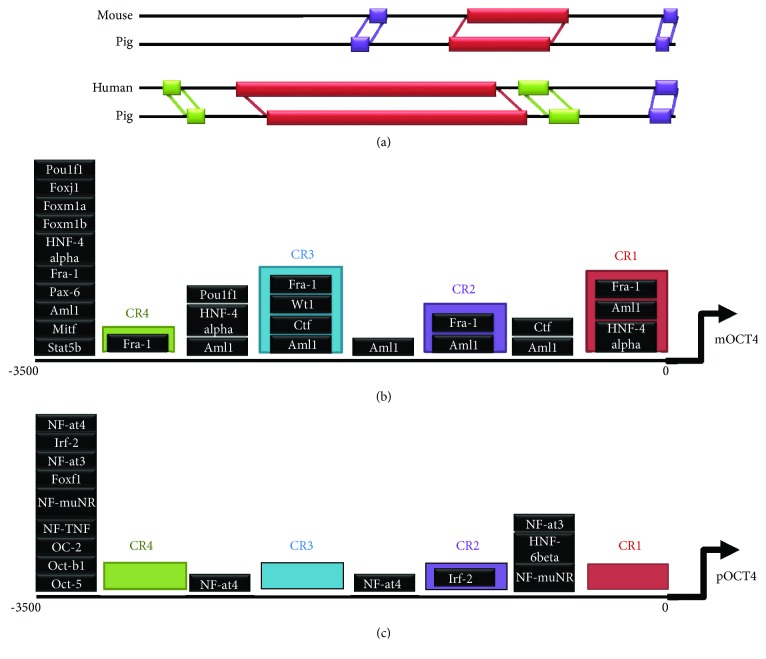
BlastN analysis and alignment of the putative transcription factor binding sites from mouse and pig using PROMO. (a) BlastN analysis between mouse and pig and human and pig. The color key for alignment scores: red: ≥200, purple: 80–200, and green: 50–80. (b) Putative transcription factor binding sites that exist in mouse sequences, but not in pig sequences. (c) Putative transcription factor binding sites that exist in pig sequences, but not in mouse sequences.

In a comparative analysis of the *Oct4* 5′ upstream genetic sequences from six mammalian species, the pig sequence contained four conserved regions, consistent with other species (Figure 3(a)). CR4 was located in -2121/-1998, CR3 was located in -1530/-1422, CR2 was located in -1146/-952, and CR1 was located in -130/-1 (Figure 3(b)). The *OCT4* conserved region was highly similar to that of many animals. The sequences of pigs and humans (CR1: 89.1%, CR2: 94.2%, CR3: 96.4%, CR4: 89.2%, and 5′ UR: 60.8%) and goats (CR1: 94.5%, CR2: 88.1%, CR3: 97.9%, CR4: 91.6%, and 5′ UR: 70.7%) were more similar than those of pigs and mice (CR1: 76.8%, CR2: 83.3%, CR3: 93.5%, CR4: 80.8%, and 5′ UR: 54.8%). The sequences from goat had the highest level of similarity with that from pig, with the exception of CR2. Throughout the *OCT4* 5′ upstream 3.5 kb nucleotide sequence, the similarity among each animal was low (Table 1). BlastN analysis showed that the sequences from pig and human were more similar than those from pig and mouse. In other words, the alignment scores from pig were higher in human than in mouse (Figure 4(a)). The putative TFBS in the 3.5 kb *Oct4* 5′ upstream region nucleotide sequences from mice and pigs also differed (Supporting Information Table 2). Some of these genes are related to pluripotency, and further research will allow us to better understand the differences in species-specific pluripotency (Figures 4(b) and 4(c)).

### 3.2. Functional Analysis of the Porcine *OCT4* Upstream Regulatory Region

**Figure 5:**
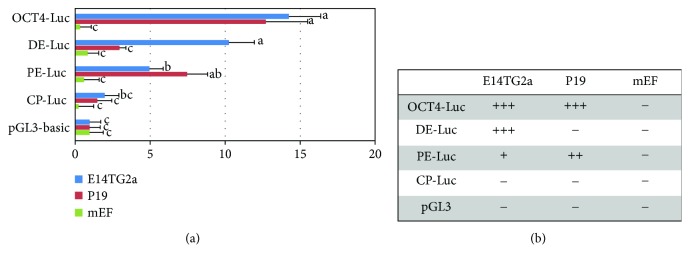
Luciferase reporter analysis of the porcine *OCT4* regulatory regions in mouse E14 ES cells, mouse P19 cells, and mouse embryonic fibroblasts. (a) Four constructs containing the different elements of the *OCT4* promoter in the pGL3-basic vector were transfected into mouse E14 ES cells, mouse P19 cells, and mouse embryonic fibroblasts. Luciferase activity was measured 48 h after transfection. Different letters indicate significant differences (*P* < 0.05). Error bars indicate the standard error of the means. (b) Reporter gene expression is tabulated on the right (compared to control): +++, high expression; ++, reduced expression; +, greatly reduced expression; and –, lack of expression in each of the different pluripotent-state cultured cell types, E14TG2a mouse embryonic stem cells, P19 mouse embryonic carcinoma cells, and mouse embryonic fibroblasts. OCT4-Luc: conserved regions 1, 2, 3, and 4 (CR1, CR2, CR3, and CR4); DE-Luc: CR1 and CR4; PE-Luc: CR1, CR2, and CR3; CP-Luc: CR1; pGL3-basic: control.

To analyze the role of the transcription regulatory elements of porcine *OCT4*, we performed a luciferase reporter assay (Figure 5). Pluripotent embryonic stem cell lines have not been derived from pig species; therefore, we transiently transfected luciferase reporter vectors containing various elements of porcine *OCT4* upstream regulatory regions into ES-E14TG2a mouse embryonic stem cells, P19 mouse embryonic carcinoma cells, and mouse embryonic fibroblasts. OCT4-Luc (p*OCT4*-pGL3) had the longest porcine *OCT4* upstream region and included all of the conserved regions (CR1, CR2, CR3, and CR4). Luciferase expression in mouse embryonic stem cells and mouse embryonic carcinoma cells expressing OCT4-Luc was significantly higher than that in mouse embryonic fibroblasts. DE-Luc (p*OCT4*-∆PE-pGL3) included CR1 and CR4. In this case, only luciferase expression in mouse embryonic stem cells expressing DE-Luc was significantly higher than that in control. In contrast, only luciferase expression in mouse carcinoma cells expressing PE-Luc (p*OCT4*-∆DE-pGL3) was significantly higher than that in mouse embryonic fibroblasts. PE-Luc contained CR1, CR2, and CR3. CP-Luc (p*OCT4*-CP-pGL3) only contained CR1, and cells transfected with this vector were not significantly different (Figure 5(a)).

Mouse embryonic stem cells expressing OCT4-Luc and DE-Luc had significantly higher luciferase expressions than cells expressing the control pGL3 vector. In contrast, mouse embryonic carcinoma cells expressing OCT4-Luc and PE-Luc had significantly higher luciferase expressions than cells expressing the control pGL3 vector. In mouse embryonic fibroblasts, luciferase expression from all constructs was not significantly different (Figure 5(b)).

### 3.3. Gene Expression Patterns in Embryonic Stem Cells and Embryonic Carcinoma Cells Separated by the Porcine *OCT4* Reporter System

**Figure 6:**
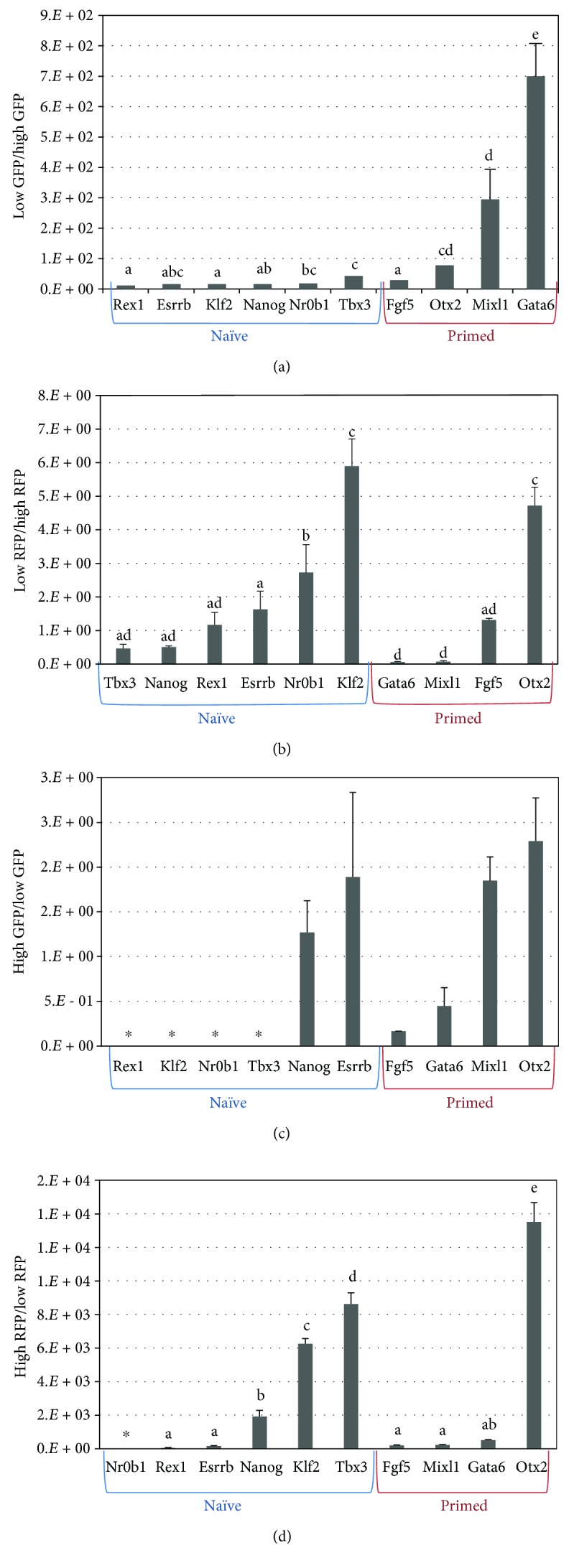
Comparison of naïve- or primed-state-related gene expression between low- and high-fluorescent cells sorted by fluorescence-activated cell sorting. Comparison of marker gene expression from naïve (*Tbx3*, *Nr0b1*, *Rex1*, *Esrrb*, *Nanog*, and *Klf2*) and primed (*Gata6*, *Mixl1*, *Fgf5*, and *Otx2*) cells using a dual-reporter assay consisting of DE-GFP (p*OCT4*-∆PE-eGFP) and PE-RFP (p*OCT4*-∆DE-DsRed2). (a) Embryonic stem cells sorted by the DE-GFP signal. Low GFP/high GFP means that the expression level of genes in GFP-low-expressing cells is divided by that in GFP-high-expressing cells. (b) Embryonic stem cells sorted by the PE-RFP signal. Low RFP/high RFP means that the expression level of genes in RFP-low-expressing cells is divided by that in RFP-high-expressing cells. (c) Embryonic carcinoma cells sorted by the DE-GFP signal. High GFP/low GFP means that the expression level of genes in GFP-high-expressing cells is divided by that in GFP-low-expressing cells. (d) Embryonic carcinoma cells sorted by the PE-RFP signal. High RFP/low RFP means that the expression level of genes in RFP-high-expressing cells is divided by that in RFP-low-expressing cells. The asterisk indicates that no values were observed.

ES-E14TG2a mouse embryonic stem cells and P19 embryonic carcinoma cells expressing DE-GFP (p*OCT4*-∆PE-eGFP) and PE-RFP (p*OCT4*-∆DE-DsRed2) were separated by FACS into groups with high and low GFP or RFP fluorescence signals. Then, the mRNA expression in each of the groups was analyzed by qPCR. In mouse embryonic stem cells, a comparison of naïve (*Tbx3*, *Nr0b1*, *Rex1*, *Esrrb*, *Nanog*, and *Klf2*) and primed (*Gata6*, *Mixl1*, *Fgf5*, and *Otx2*) state marker gene expression using a dual-reporter assay consisting of DE-GFP (p*OCT4*-∆PE-eGFP) and PE-RFP (p*OCT4*-∆DE-DsRed2) showed that naïve and primed marker expression differed in fluorescence signal between high-expressing cells and low-expressing cells. Specifically, when the expression of pluripotency genes in GFP-low-expressing cells was divided by that in GFP-high-expressing cells, GFP-low-expressing cells had relatively higher primed marker gene expressions than GFP-high-expressing cells, whereas naïve marker genes were expressed with a similar level in both cells (Figure 6(a)). It is indicated that a loss of activity of a distal enhancer gradually induces conversion of naïve pluripotency to primed pluripotency in mouse ESCs. In contrast, RFP-low-expressing cells had relatively higher expressions of several naïve marker genes and lower expressions of some primed marker genes (e.g., *mixl1* and *gata6*) than RFP-high-expressing cells. Interestingly, some primed marker genes, such as *otx2* and *fgf5*, were more highly expressed in RFP-low-expressing cells than in RFP-high-expressing cells (Figure 6(b)). There were no significant differences in the expression of all genes when P19 was divided into a DE-based GFP reporter system (Figure 6(c)). The PE-based RFP reporter system also showed irregular expression regardless of whether the genes were markers of naïve or primed states (Figure 6(d)).

## 4. Discussion

In this study, we investigated the porcine *OCT4* upstream region, provided examples of various reporter systems derived from porcine *OCT4*, and performed functional tests of the reporter system. The porcine *OCT4* upstream region has four conserved regions, two separate enhancers, several main conserved elements (e.g., the core promoter), and orthologs in other mammals [11, 17, 19, 20]. The porcine *OCT4* upstream sequences were more similar to those of human and goat than to those of mouse. This may partially explain why porcine *OCT4* expression is similar to that of human and different from that of mouse during early embryo development [27]. However, the two enhancers of porcine *OCT4* showed mechanisms similar to those previously reported in mouse. A large gap was found in the *Oct4* upstream region nucleotide sequence in both species; as such, differences in expression can occur when *Oct4* upstream region-based reporter systems constructed from one species are inserted into another species [23]. Therefore, a porcine-specific reporter system is essential for porcine stem cell research.

Enhancers with multiple cognate binding sites for various transcription factors can increase the expression of genes [28]. In particular, *Oct4* has two enhancers, a DE and a PE. They are used to produce *OCT4* in various pluripotent cells, working simultaneously or sequentially. Because different factors exist depending on the pluripotent state, *OCT4* has two enhancers to form OCT4 in different environments and is configured to operate in two different environments. The DE is a key element of the *Oct4* gene in embryonic stem cells [12, 29] and is also the most important factor in the embryonic stem cell reporter system based on the results from a previous qPCR experiment. For instance, there were ambiguities in the case of mouse embryonic stem cells separated by PE-based reporter vectors, but in the case of mouse embryonic stem cells separated by DE-based reporter vectors, the division of cell status was clear based on the intensity of the fluorescence signal. Embryonic stem cells exhibit heterogeneity during culture [30–32]; therefore, a reporter system can be used to separate more naïve cells in such mixed populations.

Embryonic carcinoma cells are primed pluripotent stem cells, and the *Oct4* PE is epiblast- and embryonic carcinoma-specific [12, 14]. Because P19 cells are known to produce *Oct4* using the PE, it is meaningless to classify P19 as a DE-based GFP signal. In fact, there was no significant difference between embryonic carcinoma cells sorted by GFP. The PE-based RFP reporter system also had problems separating embryonic carcinoma cells. In particular, the expression of genes associated with naïve and primed states in embryonic carcinoma cells sorted by fluorescent signal randomly changed. As previously reported [25], it is possible to distinguish between fluorescence patterns, but the results from the analysis of sorted cells showed that it was difficult to separate primed pluripotent cells. These results are likely due to the fact that PEs differ greatly among species [23]. Therefore, this system should be reexamined in pigs.

In summary, we conducted an analysis of the porcine *OCT4* upstream region, from which we suggested reporter systems. These reporter systems were used to distinguish and isolate mouse embryonic stem cells based on pluripotent status. Many studies of pluripotency have been conducted in mouse using *Oct4*-based reporter systems, which have been used to identify and purify pluripotent cells, as well as observe the reprogramming process [33–37]. In future experiments, we plan to introduce this reporter system into cells of porcine origin and conduct the various aforementioned experiments. Such reporter systems enable the nondestructive classification of the condition of live pluripotent cells and can be used to find a naïve-state porcine stem cell line to study species-specific pluripotency.

## Figures and Tables

**Table 1:** Alignment of the nucleotide sequences from porcine OCT4 promoter regions with those of six mammalian species (%).

Regions	Pig-mouse	Pig-vole	Pig-rabbit	Pig-cow	Pig-goat	Pig-human
CR1	76.8	76.0	83.5	66.7	**94.5**	89.1
CR2	83.3	78.7	77.4	90.1	88.1	**94.2**
CR3	93.5	88.4	94.9	**98.5**	97.9	96.4
CR4	80.8	79.7	87.0	91.5	**91.6**	89.2
5′ UR	54.8	54.1	63.8	62.0^∗^	**70.7**	60.8

The regions with the highest homology are bolded. CR1: conserved region 1; CR2: conserved region 2; CR3: conserved region 3; CR4: conserved region 4; 5′ UR: 5′ upstream region. ^∗^Gap region in the cow *Oct4* URS -2908/-2809 sequence.

## Data Availability

The data of the putative TFBS in the 3.5 kb Oct4 5′ upstream region nucleotide sequences in mouse, pig, and human used to support the findings of this study are included within the supplementary information file. All other data used to support the findings of this study are included within the article.

## Supplementary Materials

Supplementary 1 Supporting Information Table 1: primer sequences used in the gene expression analysis.

Supplementary 2 Supporting Information Table 2: the putative TFBS in the 3.5 kb Oct4 5′ upstream region nucleotide sequences in mouse, pig, and human.
