# Supplementary material for: Identification and Characterization of the OCT4 Upstream Regulatory Region in Sus scrofa
Source: Stem Cells Int. 2019 Mar 12;2019:2130973. doi: 10.1155/2019/2130973 (PMC6434273; doi:10.1155/2019/2130973)
Supplement: Supplementary 1 — Supporting Information Table 1: primer sequences used in the gene expression analysis. [file 2130973.f1.doc]

| **mRNA** | **Primer sequences (5’-3’)** |
| --- | --- |
| ACTB | F : TCCCTGGAGAAGAGCTACGA  R : CGCACTTCATGATCGAGTTG |
| Fgf5 | F : GCTCAATGATCAGAAGGAGGA  R : TCAGCTGGTCTTGAATGAGG |
| Rex1 | F : GCGGTGTGTACTGTGGTGTC  R : GACAAGCATGTGCTTCCTCA |
| Gata6 | F : TGGGAGCCATTTGGTCTATC  R : GACCTCAGATCAGCCACGTT |
| Otx2 | F : CTGGGCTGAACATTCCAGTT  R : GTCCATTTCAGGTTGCTGGT |
| Mixl1 | F : CAAGCACGCTCACAGTCAAT  R : GGAAGCTGTTTCCTGAGCTG |
| Nanog | F : TACCTCAGCCTCCAGCAGAT  R : GCAATGGATGCTGGGATACT |
| Klf2 | F : ACCAAGAGCTCGCACCTAAA  R : GTGGCACTGAAAGGGTCTGT |
| Esrrb | F : AAGTGGGGATGCTGAAGGA  R : TTCACAGAGAGTGGTCAGGG |
| Tbx3 | F : GACCATGGAACCCGAAGAAG  R : ACCATCCACCGAGAGTTGTG |
| Nr0b1 | F : CCAGGCCATCAAGAGTTTCT  R : CCCTCAATGTATTTCACGCA |

**Supporting Information Table S1. Primer sequences used for gene expression analysis.**
